# Association of Circular RNA and Long Non-Coding RNA Dysregulation with the Clinical Response to Immune Checkpoint Blockade in Cutaneous Metastatic Melanoma

**DOI:** 10.3390/biomedicines10102419

**Published:** 2022-09-27

**Authors:** Javier Oliver, Juan Luis Onieva, Maria Garrido-Barros, Miguel-Ángel Berciano-Guerrero, Alfonso Sánchez-Muñoz, María José Lozano, Angela Farngren, Martina Álvarez, Beatriz Martínez-Gálvez, Elisabeth Pérez-Ruiz, Emilio Alba, Manuel Cobo, Antonio Rueda-Domínguez, Isabel Barragán

**Affiliations:** 1 Medical Oncology Intercenter Unit, Translational Research in Cancer Immunotherapy Group, Regional and Virgen de la Victoria University Hospitals, Instituto de Investigación Biomédica de Málaga y Plataforma en Nanomedicina—IBIMA Plataforma Bionand, 29010 Malaga, Spain; 2 Facultad de Medicina, Universidad de Málaga, Campus de Teatinos s/n, 29071 Malaga, Spain; 3 Anatomy-Pathology Department, University of Málaga, 29010 Malaga, Spain; 4 Group of Pharmacoepigenetics, Department of Physiology and Pharmacology, Karolinska Institutet, 171 77 Stockholm, Sweden

**Keywords:** circRNA, cutaneous melanoma, immunotherapy, metastasis, lncRNA, ceRNA

## Abstract

Cutaneous melanoma (CM) is the most lethal form of skin cancer if it becomes metastatic, where treatment options and survival chances decrease dramatically. Immunotherapy treatments based on the immunologic checkpoint inhibitors programmed death cell protein 1 (PD-1) and cytotoxic T-lymphocyte antigen 4 (CTLA-4) constituted a main breakthrough in the treatment of metastatic CM, particularly for the achievement of long-term benefits. Even though it is a very promising therapy, resistance to primary immune checkpoint blockade (ICB) arises in about 70% of CM patients treated with a CTLA-4 inhibitor, and 40–65% of CM patients administered with a PD-1-targeting treatment. Some long non-coding RNAs (lncRNAs), and circular RNAs (circRNAs) are implicated in triggering pro- and anti-tumorigenic responses to various cancer treatments. The relationship between lncRNAs, circRNAs and ICB immunotherapy has not been explored in cutaneous metastatic melanoma (CMM). The aim of this pilot study is to evaluate the potential role of circRNA and lncRNA expression variability as pre-treatment predictor of the clinical response to immunotherapy in CMM patients. RNA-seq from 12 formalin-fixed paraffin-embedded (FFPE) samples from the metastatic biopsies of CMM patients treated with nivolumab was used to identify response-associated transcripts. Our findings indicate that specific lncRNAs and circRNAs, probably acting as competitive endogenous RNAs (ceRNAs), are involved in the regulatory networks of the immune response against metastatic melanoma that these patients have under treatment with nivolumab. Moreover, we established a risk score that yields predictions of the overall survival (OS) and progression-free survival (PFS) of CMM patients with high accuracy. This proof-of-principle work provides a possible insight into the function of ceRNAs, contributing to efforts to decipher the complex molecular mechanisms of ICB cancer treatment response.

## 1. Introduction

Melanocytes are pigment-producing cells in the skin that are derived from the neural crest during embryonic development [1]. Epidermal melanocytes can undergo a malignant tumor transformation process that leads to cutaneous melanoma (CM), which is the deadliest type of skin cancer [2]. CM is common and is increasing in incidence rates in the Western World [3]. In 2040, 510,000 new cases are expected to be diagnosed; of them, it is calculated that around 96,000 will die [3,4,5]. Both genetics and environmental risk factors have been characterized for CM. Exposure to ultraviolet radiation (UV) is the main risk factor for melanoma. UV radiation is known to generate mutations that induce cell death and the malignant transformation of melanocyte cells [6]. One of the consequences of constant exposure to UV is that melanoma has one of the highest mutation rates and mutational burdens compared to other solid malignancies [7]. Genomic studies have identified several driver genes in melanoma, such as *BRAF*, *NRAS*, *TP53*, *PTEN*, among others, as well as the relevant pathways involved in its carcinogenesis, such as the MAPK and PI3K/AKT pathways, and the cell-cycle control and telomerase programs. All of them are affected by pathologic somatic mutations in protein-coding genes [8].

Interestingly, many of these mutations arise early in the clinical process: for example, over 80% of benign nevi already have a *BRAF* mutation [9]. In more advanced stages, metastatic progression is driven by specific genomic alterations, including somatic mutations and other perturbations of genomic integrity [10,11]. Detected and treated early on, CM is highly curable. However, if CM becomes metastatic (CMM), treatment options and survival chances decrease dramatically. Immunotherapy treatments based on the immunologic checkpoint inhibitors programmed death cell protein 1 (PD-1) and cytotoxic T-lymphocyte antigen 4 (CTLA-4), such as nivolumab or ipilimumab, have been a main breakthrough in the treatment of CMM and have changed the landscape of treatment options for CM in recent years [12]. Even though it is a very promising therapy, primary immune checkpoint blockade (ICB) resistance arises in about 70% of CM patients treated with a CTLA-4 inhibitor, and 40–65% of CM patients administered with a PD-1-targeting treatment [13,14]. Several studies have proposed a variety of molecular pathways that might lead to therapy failure [14,15]. Based on them, multiple efforts are underway to determine reliable biomarkers for the prediction of immunotherapy responses, among which the predominant ones are PD-L1 expression, microsatellite instability and tumor mutational burden (TMB). Until now, only TMB has been tested as a biomarker in therapeutic trials, but it has not been found to predict clinical benefit in melanoma patients, owing to the high mutation rate of all melanoma tumors [14,16].

Long non-coding RNAs (lncRNAs) and circular RNAs (circRNAs) can act as competitive endogenous RNAs (ceRNAs) [17] and lead to a new additional post-transcriptional layer. LncRNAs participate in the control of gene expression at the epigenetic, transcriptional and post-transcriptional stages, and are important for a variety of cellular functions and molecular signaling pathways [18]. By functioning as miRNA sponges and by controlling splicing and transcription, circRNAs can affect gene expression [19]. It is becoming increasingly apparent that dysregulated lncRNAs and circRNAs are implicated in the carcinogenesis and progression of numerous cancers, acting as either oncogenes or tumor suppressors [20]. Moreover, a growing body of evidence has shown that several immune-related ceRNAs are present in the tumor microenvironment (TME), and they significantly influence immune cell infiltration and cancer-cell response to anti-PD-1 immunotherapy in different cancers [21]. However, to our knowledge, the relationship between lncRNAs, circRNAs and ICB immunotherapy has not been explored in CMM. The aim of this study is, therefore, to evaluate the potential role of circRNA and lncRNA expression variability as a clinical response predictive immunotherapy biomarker in CMM.

## 2. Materials and Methods

### 2.1. Subjects

A total of 16 metastatic melanoma patients (clinical data shown in Supplementary Table S1) treated with nivolumab donated one pre-treatment FFPE biopsy sample. The closest biopsy to the start of the ICB treatment was selected. In four cases, only the primary tumor was available, while for 12 patients, we were able to collect the metastatic lesion. Samples were collected at the Hospital Regional and Hospital Universitario Virgen de la Victoria (Málaga) from 2018 to 2019. The study followed the Declaration of Helsinki and was vetted by the Ethical Committee of Malaga, reference number 26/10/2017 with the title: “Omics integration for precision cancer immunotherapy”. For this specific analysis, we used the metastatic biopsies in order to identify biomarkers that were specific to the metastatic disease, given the scarce knowledge in the field [22] and that it is currently the most frequent indication for immunotherapy in melanoma. Responders and non-responders were defined based on RECIST v1.1 criteria: non-responders were defined as patients that progressed up to three months after the start of the treatment, and responders were the patients who maintained a partial or complete response for a year, or those that remained in treatment for at least one year.

### 2.2. Nucleic Acid Isolation

The tumor-specific area in the FFPE melanoma samples was predefined by a pathologist. Two to four 10 µm slides were dissected for nucleic acid extraction, using the microtome HM 340E (Thermo Scientific, Waltham, MA, USA). RNA was extracted with the RNeasy FFPE kit (Qiagen Dusseldorf, Germany; Ref. 73504) according to the manufacturer’s instructions.

### 2.3. Next Generation Sequencing

RNA-Seq libraries were prepared using TruSeq Stranded Total RNA Gold (Illumina; San Diego, CA, USA, EEUU; Ref. 20020598) and indexed by IDT for Illumina TruSeq RNA UD Indexes (Illumina; San Diego, EEUU Ref. 20020591). The libraries concentration was determined by the Qubit dsDNA BR kit (Thermo Scientific, Waltham, US), and the size distribution was examined by the Agilent Bioanalyzer (Santa Clara, CA 95051, USA). Paired-end reads (75bp × 2) were acquired from the Illumina NextSeq 550 platform (Illumina; San Diego, EEUU) according to the corresponding protocol.

### 2.4. Realtime PCR Validation

The expression levels of *CDR1-AS*, the most frequent circRNA, was verified by qRT-PCR using a predesigned TaqMan probe in all samples (Hs05016408_s1) (Thermo Scientific, Waltham, US) (Supplementary Figure S1).

### 2.5. lncRNA and circRNA Detection

A quality control of Fastq data from paired-end reads was performed with FastQC. Fastq files were trimmed with a cutoff of Q30. We evaluated five different pipelines to identify and quantify circRNAs reads. CIRI [23] CIRCExplorer2 [24] DCC [25], STARchip [26] and CIRIQUANT [27] were used and compared. The circRNAs sequences were annotated based on the circAtlas 2.0 database [28]. To obtain high-confidence circRNAs, we used a filtering cut-off minimum of two junction reads in at least two samples and in at least three software packages (validation strategy), which allowed a minimum of back-splice junction reads (BSJs) per circRNA. These criteria resulted in 19,030 unique circRNAs among all samples, and we used these high-confidence circRNAs for all the analyses performed in this study. With forward-splice junction reads (FSJs) and back-splice junction reads (BSJs), we used the following formula: 2*bsj/(2*bsj + fsj) to calculate the circular-to-linear transcripts ratio. LncRNA reads were identified by mapping trimmed fastq files against the reference genome GRGh38 using STAR (v 2.5.1b). Read quantification was conducted with Feature Count. LncAtlas [29] was used to annotate the lncRNAs.

### 2.6. Differential Expression Analysis

The DESeq2 pipeline with total mapped reads were used to perform the differential expression (DE) of high-confidence circRNAs and lncRNAs. The DE analysis was based on negative binomial generalized linear models, and the threshold values were set to an adjusted *p*-value < 0.1 and an absolute value of log2 (fold change) > 1.5. For the DE analysis of both circRNAs and lncRNAs, the total linear mapped read counts were used for size factor estimation.

We compared our differentially expressed of circRNAs to those reported in prior research using the circRNA disease databases circ2disease [30] circad [31] and circAtlas [28] The CSCD database [32] was used to estimate cellular localization of all detected circRNAs.

### 2.7. ceRNAs-miRNAs-mRNAs Interactions

We constructed a network composed by the circRNAs, lncRNAs and messenger RNAs (mRNAs) that were overexpressed or downregulated in relation to the response to ICB in our cohort, together with the microRNAs that interact with them. The tool Analysis of Common Targets for circRNAs (ACT) [33], which employs the miRBase [34] and miRanda [35] was used to identify microRNA (miRNA)-binding sites for the differentially expressed circRNAs. To characterize the lncRNAs and obtain the list of interactions between microRNAs (miRNAs) and the differentially expressed lncRNAs, DIANA-LncBase v2 [36] was employed. The R multiMir package [37] was used to detect the interactions between the differentially expressed messenger RNAs (mRNAs) and the miRNAs . For these interactions, we only used the subset of miRNAs that belonged to the group of miRNAs that interacted either with differentially expressed circRNAs or lncRNAs. This package combines up to seven different tools: DIANA-microT, ElMMo, MicroCosm, miRanda, miRDB, PicTar, PITA and TargetScan [38,39,40,41,42,43,44,45,46]. To improve the prediction sensitivity, only those interactions that appeared in at least five different tools were considered as a miRNA–differentially expressed mRNA pair.

Finally, we calculated the strength of the linear association between the ceRNAs (the differentially expressed circRNAs and lncRNAs) and the differentially expressed mRNAs of the network by the Pearson correlation.

### 2.8. Gene Set Enrichment and Gene Interactions Networks

The differentially expressed mRNA genes targeted by predicted miRNAs were analyzed using the Ingenuity Pathways Analysis (IPA) software version 01-20-04 (Qiagen Ingenuity Systems (www.ingenuity.com/)). Upstream regulator analysis (URA), downstream effects analysis (DEA), mechanistic networks (MN) and causal network analysis (I) prediction algorithms were used to obtain functional annotations and regulatory network analysis [47].

### 2.9. Statistics and Visualization

Statistical analyses charts and graphs were performed using R 4.0.2. The Venn Diagram R package was used to create Venn diagrams. The ComplexHeatmap R package [48] was used to create heatmaps, and the subsequent plots and graphs were created with the ggplot2 package [49]. In survival analysis, the Kaplan–Meier (KM) and log-rank tests were used to test the differences between groups. The risk score for each patient was estimated by adapting the previously described method for the estimation, using the joint expression information of the differentially expressed circRNAs and lncRNAs [50]. This joint expression was calculated with the DE value of 135 circRNAs and lncRNAs, weighted by the regression coefficients in a univariate Cox regression analysis (Equation (1)):

(1) $$\mathrm{Risk}\;\mathrm{Score}\;\left(\mathrm{RS}\right)=\overset{N}{\underset{i=1}{\sum }}({\mathrm{Expression}}_{i}{*\mathrm{Coefficient}}_{i})\;$$

where N is the number of differentially expressed circRNAs and lncRNAs, Expression-i represents the normalized expression value, and Coefficient-i is the Cox regression coefficient in the univariate model.

### 2.10. Special Case

Note on additional clinical information about patient IMK36. While this patient fulfills our criteria for non-responders, all the analyses indicate that he/she is an outlier. Soon after the start of the Nivolumab treatment, the patient presented ulcers in the legs and received antibiotic and corticoid treatment that could have inhibited the initial antitumor immune response. However, he/she has not been removed from the study to avoid reducing the sample size [51].

## 3. Results

### 3.1. Overview of circRNA and lncRNA Expression Patterns in Cutaneous Melanoma Tissues

**Figure 1:**
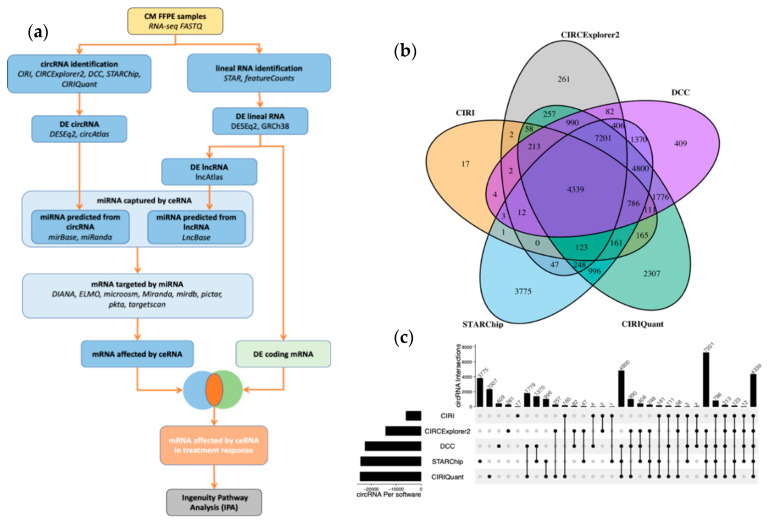
Workflow and circRNA tools comparison. (**a**) Bioinformatic workflow of the ceRNA interaction network. The pipeline is depicted from the RNA-seq fastq files to the Ingenuity Pathway Analysis. Five different software packages were employed to identify circRNAs. mRNAs affected by ceRNAs were predicted by their interaction with miRNAs. Differential expression analysis with DESeQ2 was used to decipher differences in responses to immunotherapy. (**b**) Venn diagram with the number of different cirRNAs detected by each software. (**c**) UpSet plot showing the maximum number of identified circRNAs with each software combination.

**Figure 2:**
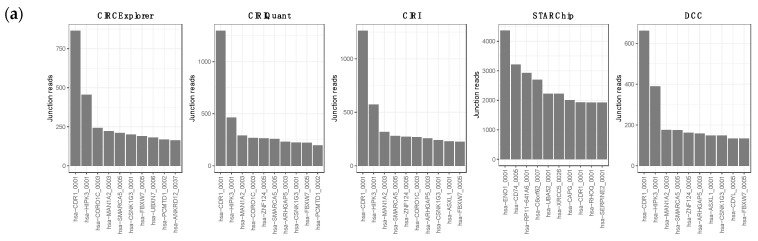

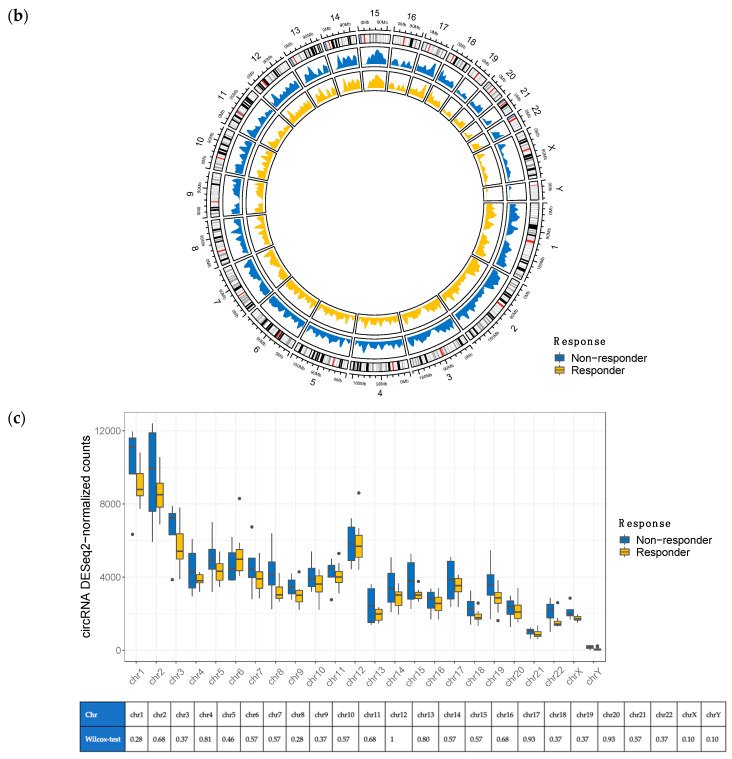
Frequencies and distribution of the circRNAs by response to ICB. (**a**) Top 10 circRNAs loci identified by the different algorithms used. (**b**) Horizontal chromosomal coverage. (**c**) Normalized count quantification per chromosome.

We analyzed the circRNA and lncRNA transcripts by RNA-seq sequencing analysis with ribosomal RNA (rRNA) depletion from the FFPE tissue of clinical CMM tumors to find aberrant expressions of these ceRNAs between responders and non-responders to the PD-1 blockade. The raw sequences were processed with five different circRNA pipelines to increase the analysis specificity and sensitivity. Only circRNAs that were found in at least three of the five pipelines were selected for further analyses (19,030 circRNAs). Both differentially expressed circRNAs and differentially expressed lncRNAs between responders and non-responders were used to build an ICB-response ceRNA network (Figure 1a). Overall, 4339 circRNA loci were detected by all tested software in metastatic tissue samples (Figure 1b,c). The top ten circRNAs generating loci were *hsa-CDR1*, *hasHIPK3*, *hsa-SMARCA5*, *hsa-CSNK1G3* and *hsa-PCMTD1*. Interestingly, *hsa-CDR1* stood as the top circRNA loci with remarkable distances to the others in four out of the five software packages. Moreover, the pattern of enrichment in non-responders was reproduced by all five (Figure 2a). The distribution of circRNAs, according to response throughout the 46 human chromosomes, indicated a similar horizontal coverage between responders (yellow line) and non-responders (blue line) (Figure 2b). However, some chromosomes, such as 1, 5, 8, 18 and 22, tend to be enriched in the circRNAs of non-responders. Furthermore, irrespective of the distribution by response, the total number of reads did not correlate with the chromosomal length. This was particularly patent in chromosome 3 and 12 (Figure 2c). Remarkably, the most significant entity was the circRNA derived from protein-coding regions (Supplementary Figure S2).

### 3.2. Differential Gene Expression of circRNAs and lncRNAs

**Figure 3:**
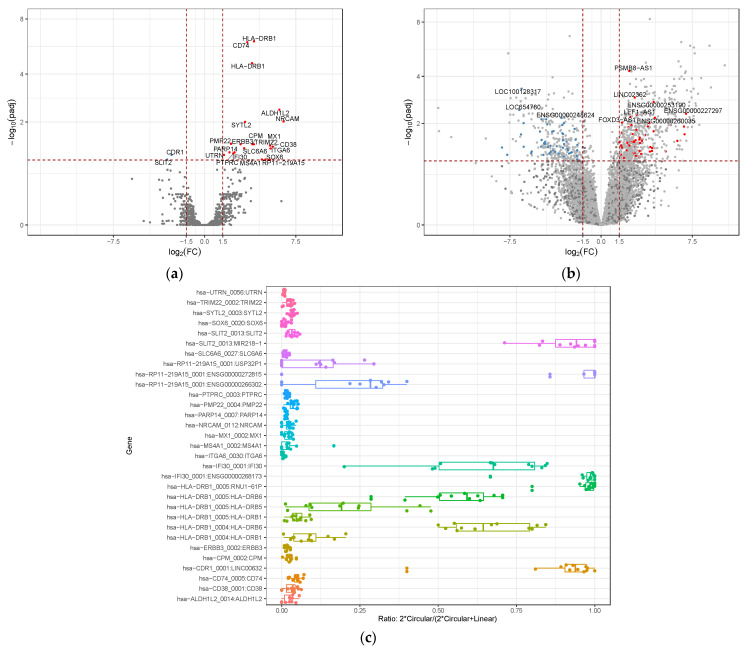

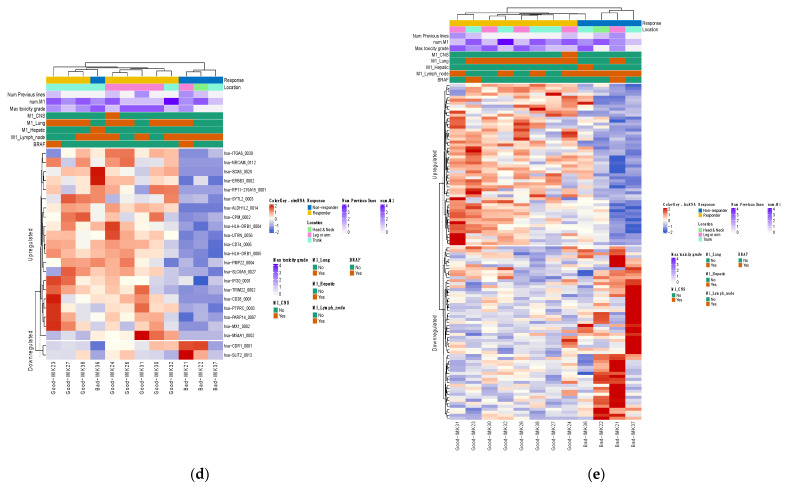
CeRNAs as biomarkers of response to ICB in metastatic melanoma. (**a**) Volcano plot with 23 circRNAs that are differentially expressed (**b**) Volcano plot with 112 lncRNAs that are differentially expressed (**c**) Ratio of circRNA vs. lineal RNA per differentially expressed circRNA loci. Y-axis lists the differentially expressed circRNAs among responders and non-responders. X-axis represents the ratio: 2*circular/(2*circular + linear), where range 0 represents deviation to linear expression and 1 maximum deviation to circRNA expression. (**d**,**e**) Expression signatures of the differentially expressed circRNAs and differentially expressed lncRNAs, respectively, separating responders and non-responders to ICB. Normalized expression values are represented against location and several clinical variables.

To analyze the expression patterns of lncRNAs and circRNAs in relation to response to immunotherapy, we identified the expression profile of dysregulated circRNAs and lncRNAs in eight responders versus four non-responders using transcriptome analysis. In the volcano map, we depict the differentially expressed circRNAs (Figure 3a) and lncRNAs (Figure 3b) with a fold-change greater than 1.5 and an adjusted p-value less than 0.1. We found 23 aberrantly expressed circRNAs, of which 21 circRNAs were upregulated and only 2 were downregulated. To further characterize the identified differentially expressed circRNAs, we retrieved data from three circRNA databases: circBase, circAltlas and the cancer-specific CircRNA Database (CSD). The annotations from the latter were particularly relevant given that almost all known circRNAs that we associated with the response are related to cancer (15 circRNAs, 68.2%). On top of that, 7 of the 23 differentially expressed circRNAs (31.8%) have been newly identified in this study. Based on the fold change, the top five most upregulated circRNAs were *hsa-ALDH1L2_0014*, *hsa-CD38_0001*, *hsa-CD74_0005*, *hsa-CDR1_0001* and *hsa-CPM_0002*.

To better understand the relation between linear and circular expression seeking other possible differences between responders and non-responders, we determined the circular–linear ratio of the differentially expressed circRNAs (Figure 3c). The inferred ratios from the RNAseq data with the formula 2*Circular/(2*Circular + Linear) showed a broad distribution ranging from 0.1 to 1. This analysis can represent the splicing preference of the loci interrogated. We observed ratios higher than 0.5 in *hsaI-SLIT2*, *hsa-RP11*, *hsa-IFI30*, *hsa-HLA-DRB1* and *hsa-CDR1*. Interestingly, *hsa-CDR1* showed one of the higher ratios and was the most relevant circRNA in term of number of counts. It was transcribed with a total of 2211 counts, distributed in 1644 vs. 567 counts between responders and non-responders, respectively.

Regarding the lncRNAs, 112 were differentially expressed with a fold change of 1.5 and an adjusted *p*-value < 0.1, from which 58 were found to be downregulated and 54 were found to be upregulated. Differentially expressed lncRNAs were annotated with LncAtlas [39].

Finally, we were able to group responders and non-responders by their expression of circRNAs and lncRNAs. Hierarchical clustering analysis showed responders discrimination among responders and non-responders for both types of RNAs (Figure 3d,e).

### 3.3. Competitor Endogenous RNA Network (ceRNA Network)

**Figure 4:**
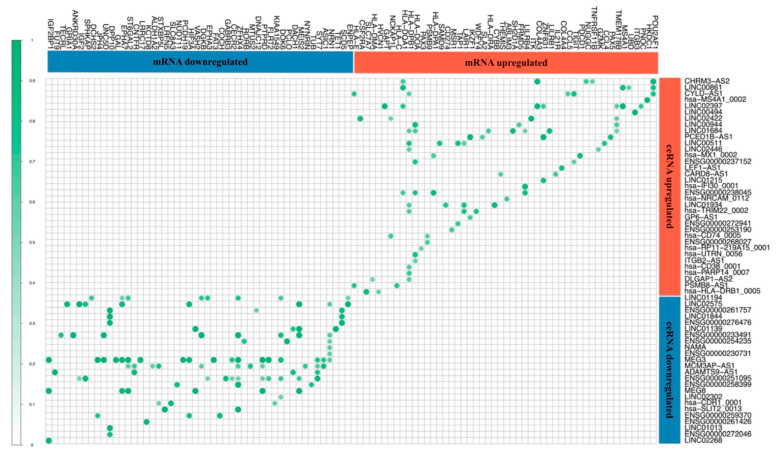
Correlation ceRNA network. Lineal correlation of expression of the differentially expressed ceRNAs and the differentially expressed mRNAs that belong to the ceRNA interaction network.

In order to understand the role of the differentially expressed circRNAs and lncRNAs as post-translational regulators in the context of resistance to ICB, a ceRNAs Network was built that included 69 lncRNAs with a miRNA interaction (36 upregulated and 33 downregulated; the 43 missing lncRNAs that add up to the total 122 differentially expressed lncRNAs do not have any miRNA interaction) and 23 circRNAs (21 upregulated and 2 downregulated). Two additional layers complemented the network. One of them consisted of 537 target miRNAs from the miRBase with strongly predicted binding sites to our differentially expressed circRNAs and lncRNAs. Of them, the ones showing the highest prediction values were *Let-7e-5p*, *miR-1285-3p*, *miR-6757-3p*, *miR-877-3p* and *miR-3689d*. The second additional component comprised 154 differentially expressed mRNAs among the responders and non-responders that showed interactions with miRNAs predicted to bind with the differentially expressed ceRNAs. Furthermore, the statistical correlation between the differentially expressed mRNAs regulated by these miRNAs, and the differentially expressed ceRNAs, showed that all correlations were direct, reinforcing the notion of the ceRNAs’ inhibitory role on miRNAs’ action (Figure 4). We also identified several major putative regulators, such as *LINC00861*, *CHRM3-AS2*, *MEG3* and *RP11−115D19.1*, which correlated with multiple mRNAs, as well as three mRNAs that we speculate can be regulated by two or more ceRNAs in the context of melanoma resistance to nivolumab: *ICOS, PAX3, HLA−DOA* and *HLA−DPB1*.

### 3.4. IPA Functional Enrichment Analysis Based on the ceRNA Network

**Figure 5:**
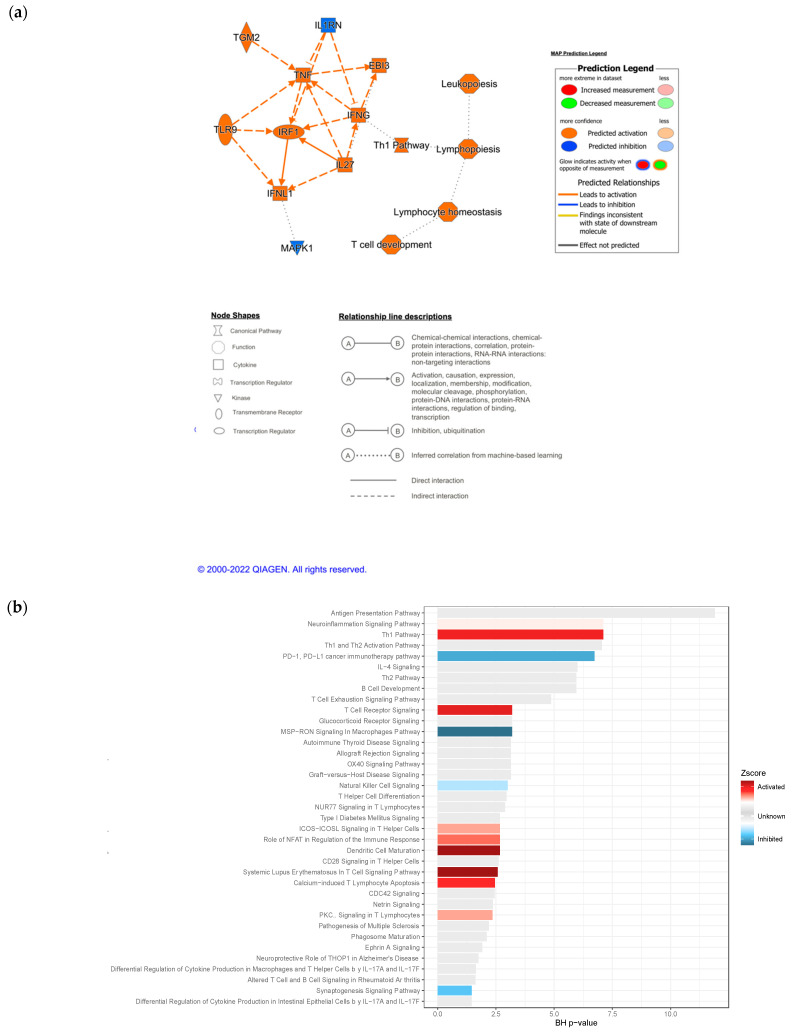

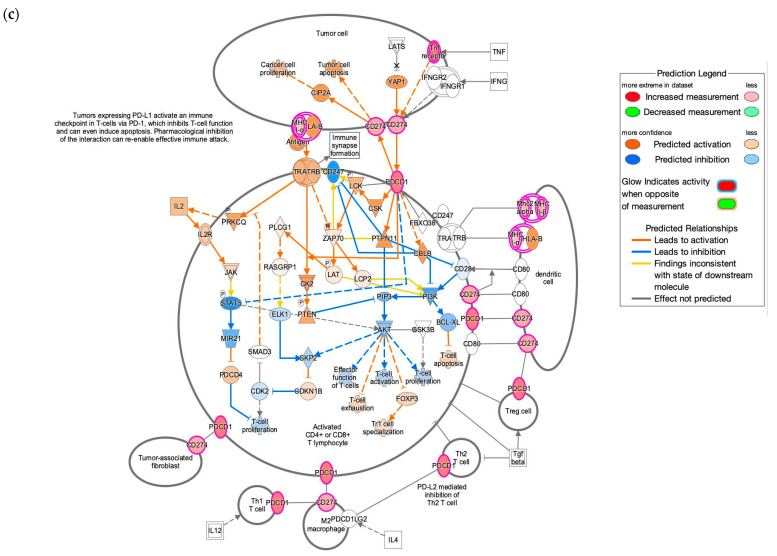
Specific immunological perturbation associated with the ceRNA network. (**a**) Influence network showing the activation or inhibition of the key molecules and pathways defined by the ceRNAs. (**b**) Pathways enriched and influenced by the ceRNA dysregulation according to response to ICB. (**c**) Activated and inhibited genes and pathways of the PD1–PDL1 axis in the context of ceRNA dysregulation.

In order to gain more quantitative and qualitative insight into the mechanism of the putative regulation of ICB response by ceRNAs, we characterized the biofunctions and diseases associated with the differentially expressed mRNAs that interacted with the differentially expressed ceRNAs, as well as the downstream and upstream modulators. Influence network analysis shows that the DE of the interactome key molecules led to activity changes, mostly in immunological processes. Activated molecules included TNF, IRF, IL27, TLR9, EIB3, TGM2 and IFNG, and inhibited ones included MAPK1 and IL1RN (Figure 5a). Consistently, almost all the enriched functional categories were related to the immune response, and even specifically to the PD-1–PD-L1 cancer immunotherapy pathway, the target axis of nivolumab, as show in Figure 5b. This figure depicts the enriched pathways and the direction based on the Z-score: the most activated pathways of responders were the TH1 pathway, the T-cell receptor signaling pathway, the ICOS-ICOS-L signaling pathway in T-helper cells, the role of NFAT in the regulation of the immune response, dendritic cell maturation, and calcium-induced T lymphocyte apoptosis. On the other hand, pathways with a Z-score predicting pathway inhibition were the natural-killer cell-signaling pathway, the synaptogenesis signaling pathway and PD-1, as well as PD-L1 cancer immunotherapy, together with MSP–RON signaling in the macrophages pathway. Regarding molecules, the most relevant molecules annotated for these molecular functions were CCL5, CD6, CSF2RA, HLA-B HLA-DRA, HLA-E, ICOS, IKZF1, IL12RB1, IL12R, LAIR1, LILRB2, LILRB4, MS4A1, PDCD1, TBX21 and UBD. Even though the Z-score of relevant pathways for the response indicated an inhibition of the axis, it is important to evaluate the individual contribution of the differentially expressed genes in our dataset. Indeed, in Figure 5c, we can observe that the relevant immune response elements of the PD−1-PD−L1 axis, such as those coding for the receptor of TNF (TNFR), IFNγ, MHC1α, β and PD1, are upregulated in responders to nivolumab (Figure 5c).

Next, by using the IPA upstream regulator analysis tool applied on the ceRNA network, we can predict upstream molecules and provide a mechanistic network that could explain the observed changes in gene expression. Interestingly, we observed the following activated transcription regulators: SOX11, IRF1, NLRC5 and SMRACA4. NEUROG1 was found uniquely inhibited. Regarding cytokines, IL1RN and IL13 were inhibited and IL27, IFLN1, TNF, IFNG and EBI3 (Supplementary Figure S3) were predicted to be activated. Other altered upstream regulators were TLR9 and TGM2, which were activated, and SAFB, SAFB2, RARA and ESR1, which were found inhibited.

**Figure 6:**
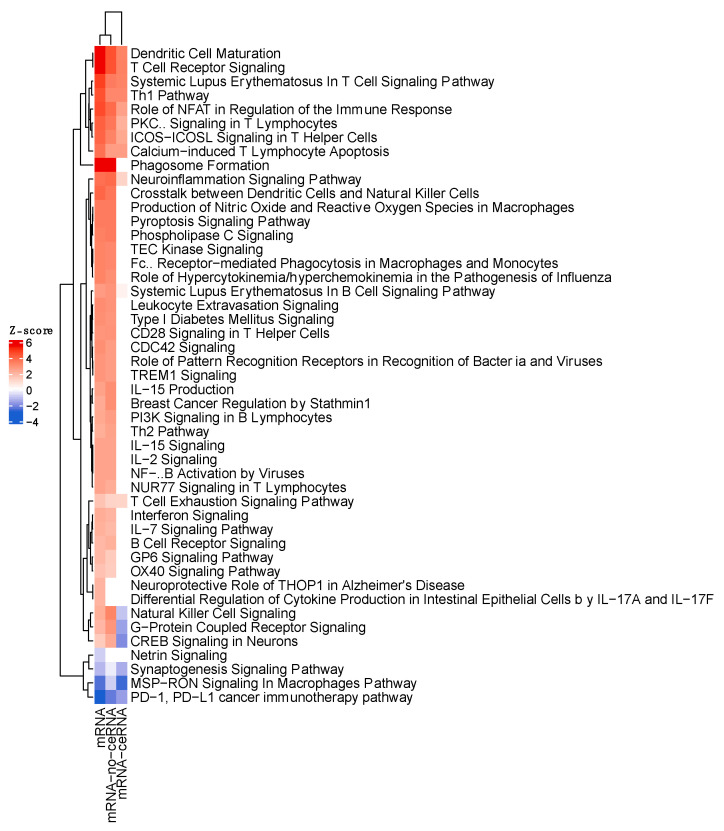
Role of ceRNA as modulators of the ICB resistance. Comparison of the pathways’ aberration according to absence or presence of mRNAs associated with ceRNAs.

Furthermore, to characterize the specific role of ceRNAs in the regulation of the mechanism of resistance to ICB, we sought to identify the pathways aberration related specifically to response genes that are correlated with such regulators. For this, we generated Z-scores of IPA canonical pathways of all differentially expressed mRNAs (DEmRNA) vs. the DEmRNA correlated with ceRNAs (DEmRNA-ceRNA) and vs. the differentially expressed mRNAs not associated with ceRNA (DEmRNA-noceRNA) (Figure 6). It is very important to note that there are three pathways that denote an opposite activation profile compared with all or non-ceRNA-related mRNAs. One of them is the natural-killer cell-signaling pathway. Additionally, the Z-score of PD−1, PD−L1 cancer immunotherapy was higher when the pathway was defined by the expression differences of the mRNAs related to ceRNAs. Moreover, ceRNAs seem to be involved in a fraction of the mechanisms associated with drug response. This indicates that ceRNAs may modulate specific processes of ICB resistance independently of other regulators that they can synergize or oppose.

Moreover, the gene ontologies and pathways were determined with IPA. The statistical overrepresentation test was used to find the enriched GO terms and pathways by matching our gene list with the human genome. The most relevant biological processes were as follows: leukopoiesis, lymphopoiesis, cell development and lymphocyte homeostasis (GO:0002521, GO:0030098, GO:0046650, GO:0002260). Supplementary Figure S4 represents the top enriched ontologies for disease and disorders, molecular and cellular function, and the physiological system and physiology. The enriched ontology terms were mainly related to cancer, cellular growth and cellular proliferation, as well as immunological conditions. The most enriched molecular and cellular functions were cellular development, cellular growth and cell-to-cell signaling and proliferation. The most enriched physiological systems were the hematological system development and function system, the lymphoid tissue structure and development system and the immune cell-trafficking system. Like the GO term results, the KEGG pathway enrichment analysis identified Axon guidance, the T-cell receptor signaling pathway, natural-killer cell-mediated cytotoxicity, the ErbB signaling pathway and the Fc epsilon RI signaling pathway as the enriched pathways in the interactome.

### 3.5. Prognostic Risk Score Using the Differentially Expressed ceRNA

**Figure 7:**
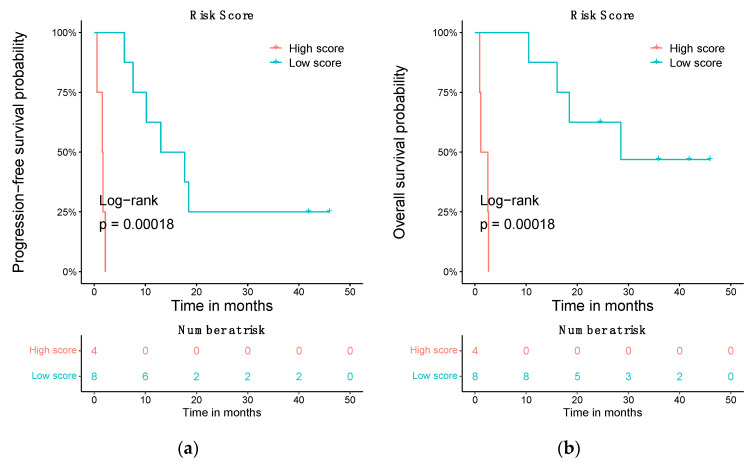
Prognostic value of the ceRNA signature. (**a**) Association of the ceRNA Risk score based on gene expression levels with OS in cutaneous metastatic melanoma. (**b**) Stratification of the patients according to PFS using the ceRNA-based risk score.

A survival risk score based on the basal gene expression of 135 differentially expressed ceRNAs categorized the patients in two groups: high and low risk. A high value risk score was linked with a ceRNA signature of worse overall survival, whereas a low value was associated with an ceRNA expression pattern of better survival. Patients considered low risk had a greater significant overall survival (OS) with a log-rank *p*-value of 0.00018. The low-risk patients’ median OS was of 28.49 months (95% CI, 18.46—NR), while patients considered high risk had a median OS of 1.84 months (95% CI, 0.92—NR) (Figure 7a). Concerning progression-free survival (PFS), this score is able to predict which patients will be in the high- or low-risk categories with a log-rank *p*-value of 0.00018 (Figure 7b). The median PFS of low-risk patients was of 15.34 months (95% CI, 10.20—NR), while high-risk patients had a median PFS of 1.64 months (95% CI, 0.53—NR).

## 4. Discussion

In an attempt to unmask new players in the control of ICB resistance mechanisms, we characterized the association of RNA species of recent annotation with the response to ICB in metastatic CM patients. Therefore, we performed transcriptome analysis of bulk metastatic melanoma tissue to generate information from both tumor and immune cells from a metastatic niche. Our hypothesis was that circRNAs and lncRNAs could regulate the response to ICBs by exerting a sponge function that inhibited specific miRNAs as ceRNAs. In order to test this, we integrated miRNAs that were targets of differentially expressed ceRNAs and targeted of differentially expressed mRNAs from our dataset. The resulting selection of differentially expressed mRNAs targeted by those miRNAs was subjected to a correlation test with ceRNAs. Consistent with our hypothesis, the expression correlations were always direct, suggesting that the increased expression of ceRNAs was aligned with the increased expression of the mRNA, putatively through the inhibition of the corresponding miRNA. Since miRNAs’ expression information is absent from our dataset, our conclusions cannot include the direct association with the miRNAs. In line with several previous reports that have highlighted the importance of immune function in the process of melanoma metastasis and ICB response [52,53,54,55], our results show a profound influence of the dysregulation of these non-coding RNAS (ncRNAs) on the activation or inhibition of key anti-tumor immunological processes, such as the Th1, natural-killer cell-signaling, T-cell receptor signaling or the PD-1–PD-L1 cancer immunotherapy pathways. Interestingly, the expression perturbation of the responders was associated with an increasing expression of PD-1 and PD-L1 (CD274). The identification of these ceRNAs as regulators of PD-1 and PD-L1 expression is important for understanding the interindividual variation in this axis, which holds the first FDA-approved marker for ICB [53].

On the other hand, the identification of the pattern of pathway aberration that was exclusive to the ceRNAs-associated genes unmasked a modulatory role of the ceRNAs for a subset of specific resistance pathways. This finding implies that ceRNAs can either refine or oppose the effects on drug response processes. This is particularly important for two pathways that are intrinsically related to the ICB response: the natural-killer cell signaling pathway and PD-1–PD-L1 cancer immunotherapy. The dysregulation of the ceRNAs in responders is related to the inhibition of the natural-killer cells, while it ameliorates the inhibition of the PD-1/PD-L1 axis.

With all these observations, it can be envisaged that the major disruption of immunological anti-tumor pathways related to ceRNAs and observed in responders denote the crucial role that the tumor immune microenvironment (TIME) plays in the treatment response to ICB. Some lines of evidence indicate that tumor cells can modify the TIME by recruiting immunosuppressive cells, and both circRNAs and lncRNAs are tools that tumor cells can use via extravesicular particles to obtain a favorable TIME, consequently leading to treatment failure. Interestingly, previous reports, regarding both hepatocellular carcinoma and pancreatic cancer, associated specific circRNAs with the response to targeted therapy and linked them to natural killer cell dysregulation [54]. With IPA analyses, we can precisely predict functional regulatory networks from gene expression data and assign a significance score to each network based on how well it fits the database’s set of focus genes [47]. Two of the main molecules highlighted by the ceRNA network (Figure 5a) are TNF and IFNG. Recently, the overexpression of TNF, IFNG and IL2, among other molecules, have been reported as key molecules that may enhance melanoma progression through activating the JAK–STAT signaling pathway [55]. Moreover, other studies indicate that both TNF and IFNG are directly linked with the high density, T-cell infiltration and cytotoxicity of cytotoxic T-cell (CTL) functions [56]. This process has been recently characterized by Weigelin B. et al., 2021, whose findings suggested that CTL-mediated apoptosis induction is not a one-size-fits-all process, and the most common mechanism of tumor-cell eradication by antigen-specific CTL is the accumulation of sublethal damages [57]. Additionally, other studies have shown a strong positively correlation between TNF and PD-L1 expression and poor prognosis [58]. Based on our dataset, we speculate that some of the identified differentially expressed mRNAs of the TNF ligand family (*TNFSF8*, *TNFRSF9*, *TNFRSF17*, *TNFRSF13B*, *TNFRSF12A* and *TNFRSF11B*) may affect the CTL activity and the lymphocyte infiltration. This supports the relevance of TNF in response to ICB in CMM and contributes to the support of the concept of combining therapies based on anti-TNF and anti-PD-1 in CMM.

With regards to the specific ceRNAs identified in this study, *CDR1* circRNA stands out as a potentially implementable biomarker of response. Previous studies have highlighted the relevance of *CDR1* in cancer, particularly in the metastatic melanoma process. The main biological process identified to date is sponging *miRNA-7*, which is well established as a cancer progression marker. Recently, Hanniford D. et al. 2020 described a more complex regulation of this region via the epigenetic silencing of the lncRNA *LINC00632* [59]. In our study, we observed that *CDR1* is one of the most relevant differentially expressed circRNAs in terms of abundancy and differential expression in non-responders. Moreover, we observed that the responders tend to have a more homogeneous *CDR1* expression, indicating that *CDR1* seems to be dysregulated in most of the non-responders. Further studies are needed to validate *CDR1* for predicting and monitoring treatment response.

Finally, this work has found another utility as a generator of a prognosis prediction model in the context of responses to ICB in metastatic CM. A prognostic risk score has been created for the signature of ceRNAs and used to stratify patients at a high and low risk regarding OS and PFS. The application of this score can be used to predict these outcomes. To our knowledge, this the first time that a differentially expressed ceRNA’s signature could be associated with a prognosis of any ICB treatment.

This study is a proof-of-principle work which develops a highly reliable bioinformatic pipeline to identify circRNAs and lncRNAs, as well as expression survival scores. Despite the small sample size and the lack of available validation datasets due to the novelty of the approach, our results and conclusions are compatible with the ceRNA hypothesis and generate a unique response signature that warrant further validation in independent cohorts.

All in all, this work provides a novel insight into the modulators of ICB resistance and implies the existence of new players to be considered as prognosis biomarkers and targets to counteract resistance in ICB-treated cutaneous melanoma.

## 5. Conclusions

In the present study, we characterized the ceRNAs in metastatic melanoma patients treated with ICB to explore the biological role of the ceRNA network on the responses. Our exploratory analysis revealed that ceRNAs can modulate specific ICB resistance processes; therefore, they need to be considered in the complex regulatory scenario of the TIME interactions. Finally, the definition of a Risk score based on the ceRNA expression signature constitutes a potentially useful tool for predicting prognoses in the context of ICB treatment in metastatic CM.

## Data Availability

The data that support the findings of this study are available from the corresponding author, I.B., upon reasonable request.

## Supplementary Materials

The following are available online at https://www.mdpi.com/article/10.3390/biomedicines10102419/s1, Figure S1: RT-PCR validation of *CDR1*. (a) Correlation between ∆Ct obtained by real time PCR and TPM generated by RNAseq. (b) ∆Ct of responders and non-responders; Figure S2: Exon type of circRNAs. Mapping of circRNAs indicates that 98.3% of circRNAs identified are exon-exon type; Figure S3: Interaction networks and upstream molecules. (a) Interaction networks of the main molecules of the differentially expressed mRNAs of the ceRNA network predicted by IPA. (b) Main relevant acti-vated upstream molecules are IL27 and EBI3, while SAFB is an important inhibited upstream reg-ulator associated to our ceRNA network. Figure S4. Top enriched IPA ontologies and pathways of the molecular and cellular function and the physiological system and physiology. Table S1. Clinical characteristics of 12 CMM treated with immunotherapy.
