# Supplementary material for: Association of Circular RNA and Long Non-Coding RNA Dysregulation with the Clinical Response to Immune Checkpoint Blockade in Cutaneous Metastatic Melanoma
Source: Biomedicines. 2022 Sep 27;10(10):2419. doi: 10.3390/biomedicines10102419 (PMC9599084; doi:10.3390/biomedicines10102419)
Supplement: Supplementary file 1 [file biomedicines-10-02419-s001.zip › biomedicines-1828639-supplementary.pdf]

biomedicines

MDPI

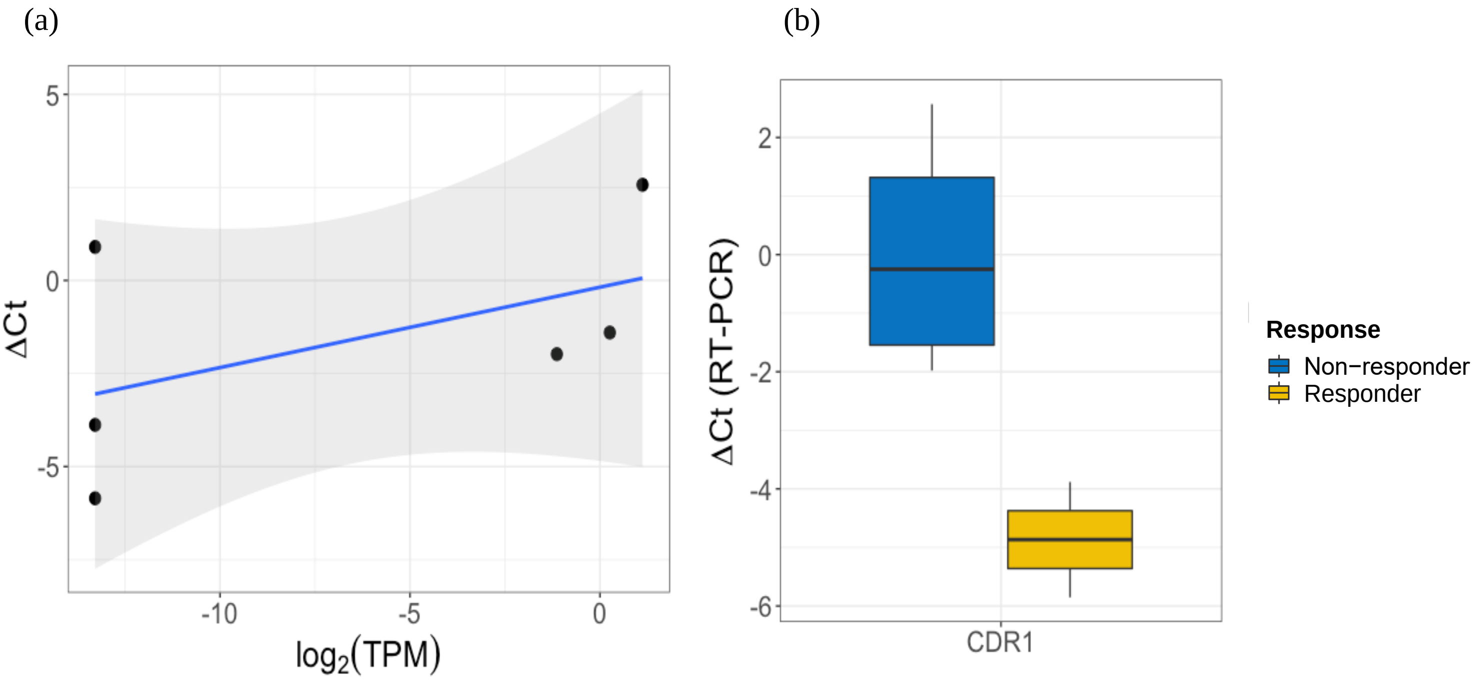


**Supplementary Figure S1. RT-PCR validation of *CDR1*.** (**a**) Correlation between ΔCt obtained by real time PCR and TPM generated by RNA-seq. (b) ΔCt of responders and non-responders.

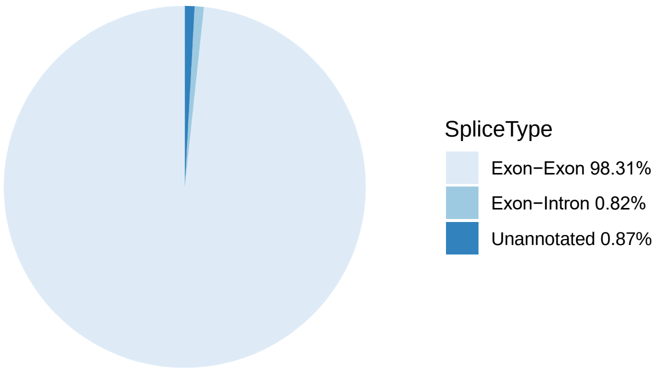


**Supplementary Figure S2**. CircRNAs exon types. Mapping of circRNAs indicates that 98.3% of identified circRNAs are exon-exon type.

(a)

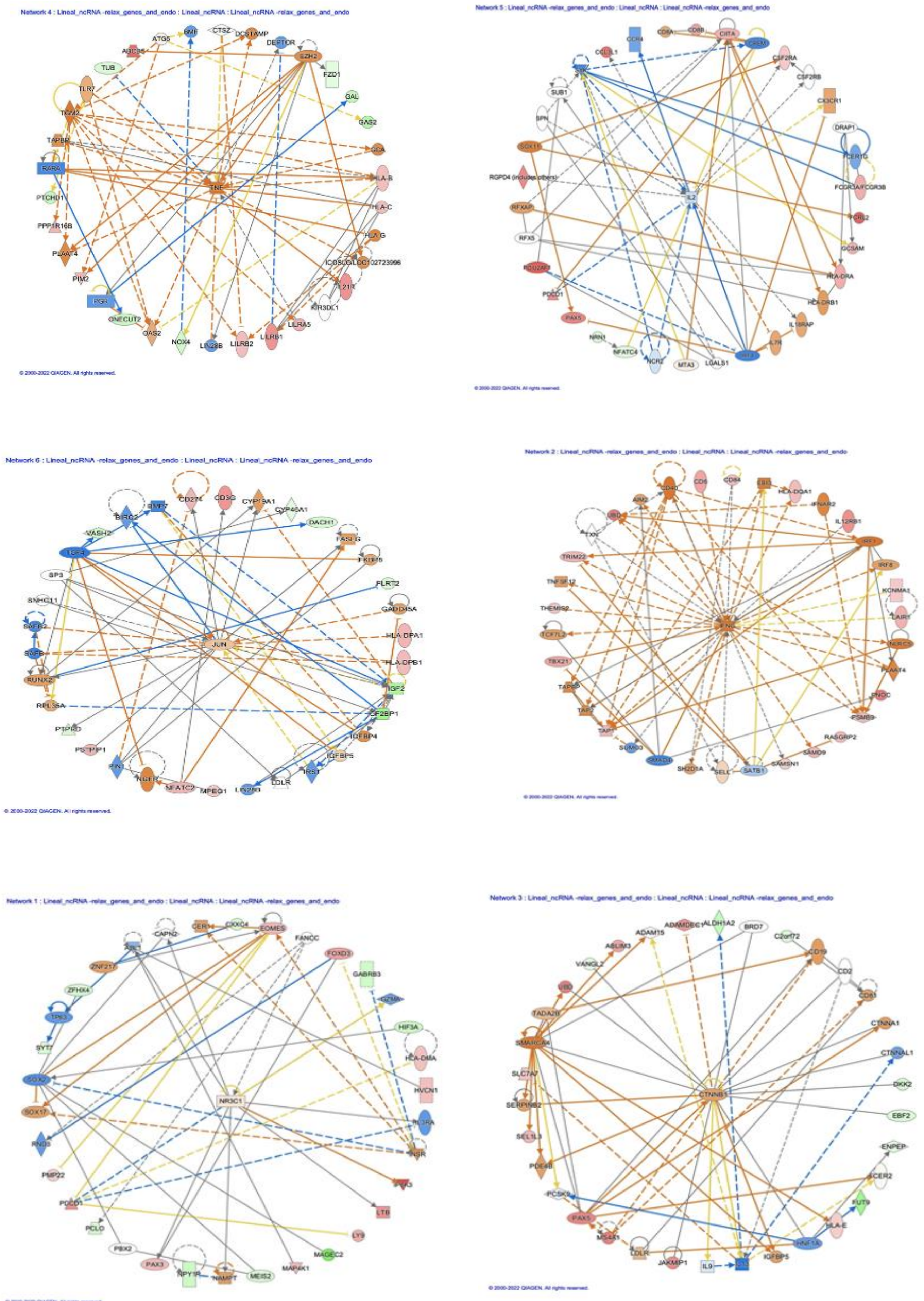
Network 4 : Lineal_ncRNA -relax_genes_and_endo : Lineal_ncRNA : Lineal_ncRNA -relax_genes_and_endo
© 2000-2022 QIAGEN. All rights reserved.
Network 5 : Lineal_ncRNA -relax_genes_and_endo : Lineal_ncRNA : Lineal_ncRNA -relax_genes_and_endo
© 2000-2022 QIAGEN. All rights reserved.
Network 6 : Lineal_ncRNA -relax_genes_and_endo : Lineal_ncRNA : Lineal_ncRNA -relax_genes_and_endo
© 2000-2022 QIAGEN. All rights reserved.
Network 2 : Lineal_ncRNA -relax_genes_and_endo : Lineal_ncRNA : Lineal_ncRNA -relax_genes_and_endo
© 2000-2022 QIAGEN. All rights reserved.
Network 1 : Lineal_ncRNA -relax_genes_and_endo : Lineal_ncRNA : Lineal_ncRNA -relax_genes_and_endo
Network 3 : Lineal_ncRNA -relax_genes_and_endo : Lineal_ncRNA : Lineal_ncRNA -relax_genes_and_endo
© 2000-2022 QIAGEN. All rights reserved.

(b)

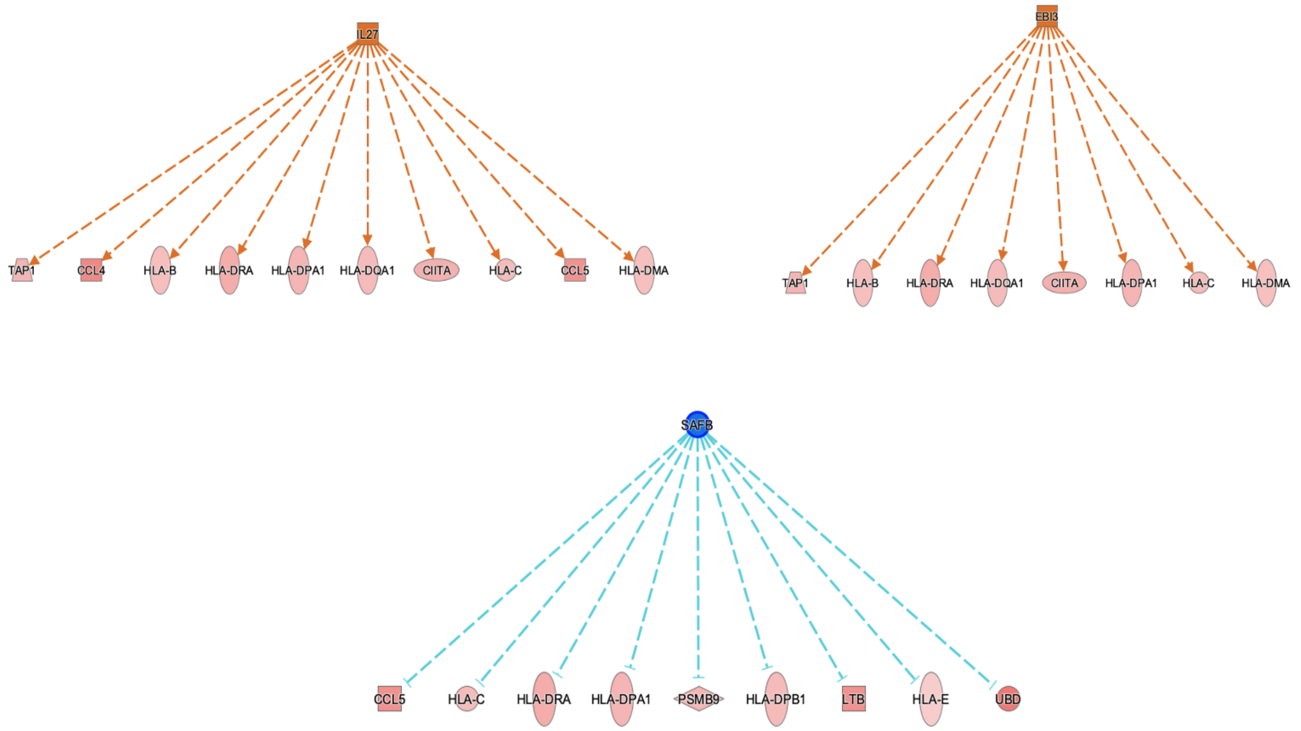


**Supplementary Figure S3.** Interaction networks and upstream molecules. **(a)** Interaction networks of the main molecules of the differentially expressed mRNAs of the ceRNA network predicted by IPA. **(b)** Main relevant activated upstream molecules are IL27 and EBI3, while SAFB is an important inhibited upstream regulator associated to our ceRNA network.

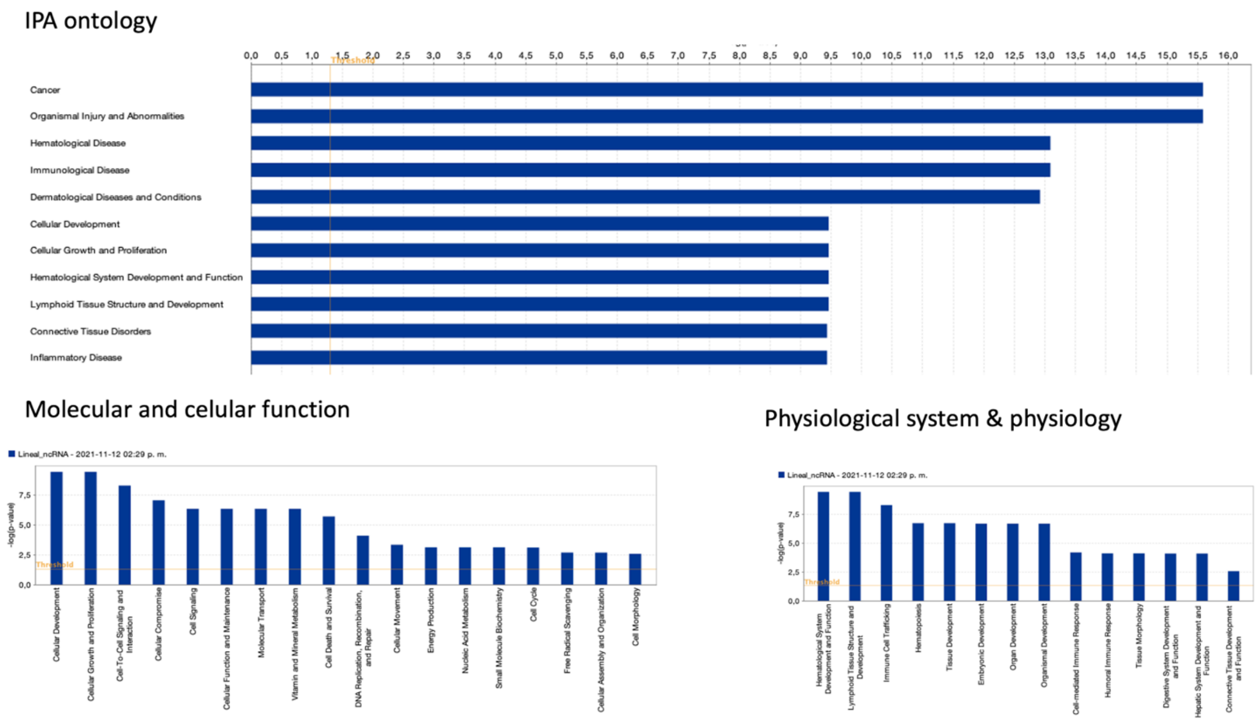


**Supplementary Figure S4**. Top enriched IPA ontologies and pathways of the molecular and cellular function and the physiological system and physiology.

**Supplementary Table S1.** Clinical characteristics of 12 CMM patients treated with immunotherapy.

| Variables | Value | Responders | Non-responders | Lost Values |
|---|---|---|---|---|
| **Sex** | | | | 0 (0%) |
| Female | 6 (50%) | 5 (62.5%) | 1 (25%) | |
| Male | 6 (50%) | 3 (37.5%) | 3 (75%) | |
| **Stage at diagnosis** | | | | 1 (0.08%) |
| Stage I | 1 (9.09%) | 1 (14.29%) | 0 (0%) | |
| Stage II | 4 (36.36%) | 2 (28.57%) | 2 (50%) | |
| Stage III | 4 (36.36%) | 4 (57.14%) | 0 (0%) | |
| Stage IV | 2 (18.18%) | 0 (0%) | 2 (50%) | |
| **Diabetes** | | | | 0 (0%) |
| No | 10 (83.33%) | 7 (87.5%) | 3 (75%) | |

| | | | | |
|---|---|---|---|---|
| Yes | 2 (16.67%) | 1 (12.5%) | 1 (25%) | |
| **Dyslipidemia** | | | | 0 (0%) |
| No | 9 (75%) | 5 (62.5%) | 4 (100%) | |
| Yes | 3 (25%) | 3 (37.5%) | 0 (0%) | |
| **Hypertension** | | | | 0 (0%) |
| No | 4 (33.33%) | 2 (25%) | 2 (50%) | |
| Yes | 8 (66.67%) | 6 (75%) | 2 (50%) | |
| **Status** | | | | |
| Alive | 4 (33.33%) | 4 (50%) | 0 (0%) | |
| Dead | 8 (66.67%) | 4 (50%) | 4 (100%) | |
| **Overall survival time** | 17.26 [0.92–45.97] | 26.49 [10.52–45.97] | 1.84 [0.92–3.28] | 0 (0%) |
| **Progression** | | | | |
| No | 3 (25%) | 3 (37.5%) | 0 (0%) | |
| Yes | 9 (75%) | 5 (62.5%) | 4 (100%) | |
| **Progression-free survival time** | 8.98 [0.52–45.97] | 15.74 [5.9 – 45.97] | 2.08 [0.52–2.33] | 0 (0%) |
| **Stage prior to ICB treatment** | | | | |
| **Stage I** | 0 (0%) | 0 (0%) | 0 (0%) | |
| **Stage II** | 0 (0%) | 0 (0%) | 0 (0%) | |
| **Stage III** | 0 (0%) | 0 (0%) | 0 (0%) | |
| **Stage IV** | 11 (100%) | 8 (100%) | 4 (100%) | 0 (0%) |
| **No. metastasis** | 2 [1–4] | 2.5 [1–4] | 2 [1–3] | 0 (0%) |
| **Lung metastasis** | | | | 0 (0%) |

| | | | | |
|---|---|---|---|---|
| No | 4 (33.33%) | 1 (12.5%) | 3 (75%) | |
| Yes | 8 (66.67%) | 7 (87.5%) | 1 (25%) | |
| **Lymph node metastasis** | | | | 0 (0%) |
| No | 4 (33.33%) | 4 (50%) | 0 (0%) | |
| Yes | 8 (66.67%) | 4 (50%) | 4 (100%) | |
| **LDH first metastasis (U/L)** | 164 [107–498] | 215 [164–498] | 144 [107–153] | 5 (0.42%) |
| **LDH previous to IT (U/L)** | 235.5 [107–498] | 260 [208–498] | 191 [107–249] | 4 (0.33%) |
| **Lymphocytes previous to IT (counts × $10^6$/L)** | 1410 [370–2300] | 1580 [370–2300] | 1100 [500–1520] | 2 (0.17%) |
| ***BRAF*** | | | | 0 (0%) |
| Wild-type | 10 (83.33%) | 7 (87.5%) | 3 (75%) | |
| Mutated | 2 (16.67%) | 1 (12.5%) | 1 (25%) | |
| **No. Previous lines** | 0 [0–2] | 0 [0–2] | 0 [0–1] | 0 (0%) |
| **Age at start IT** | 62.74 [19.1–74.38] | 68.91 [19.1–74.38] | 55.01 [41.32–68.72] | 0 (0%) |
| **Toxicity IT** | | | | 0 (0%) |
| No | 3 (25%) | 1 (12.5%) | 2 (50%) | |
| Yes | 9 (75%) | 7 (87.5%) | 2 (50%) | |
